# Supplementary material for: Influence of Proteins on Bioaccessibility of α-Tocopherol Encapsulation within High Diacylglycerol-Based Emulsions
Source: Foods. 2023 Jun 25;12(13):2483. doi: 10.3390/foods12132483 (PMC10341297; doi:10.3390/foods12132483)
Supplement: Supplementary file 1 [file foods-12-02483-s001.zip › foods-2419177-supplementary.pdf]

Supplementary material

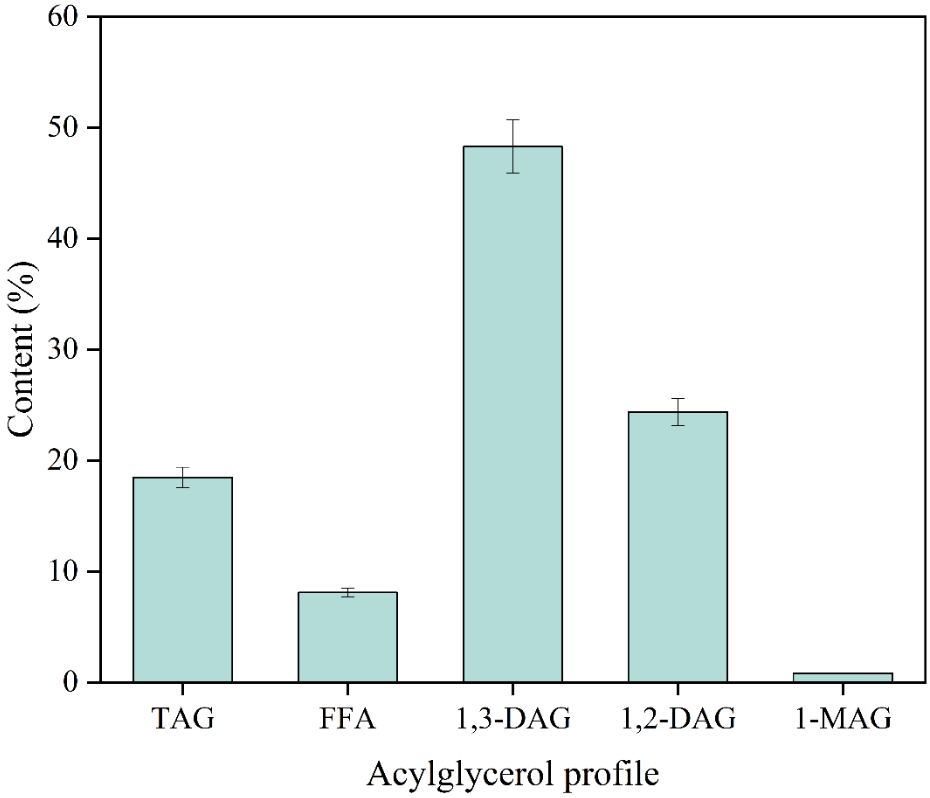

Figure.S1. Lipid composition of Olive diacylglycerol

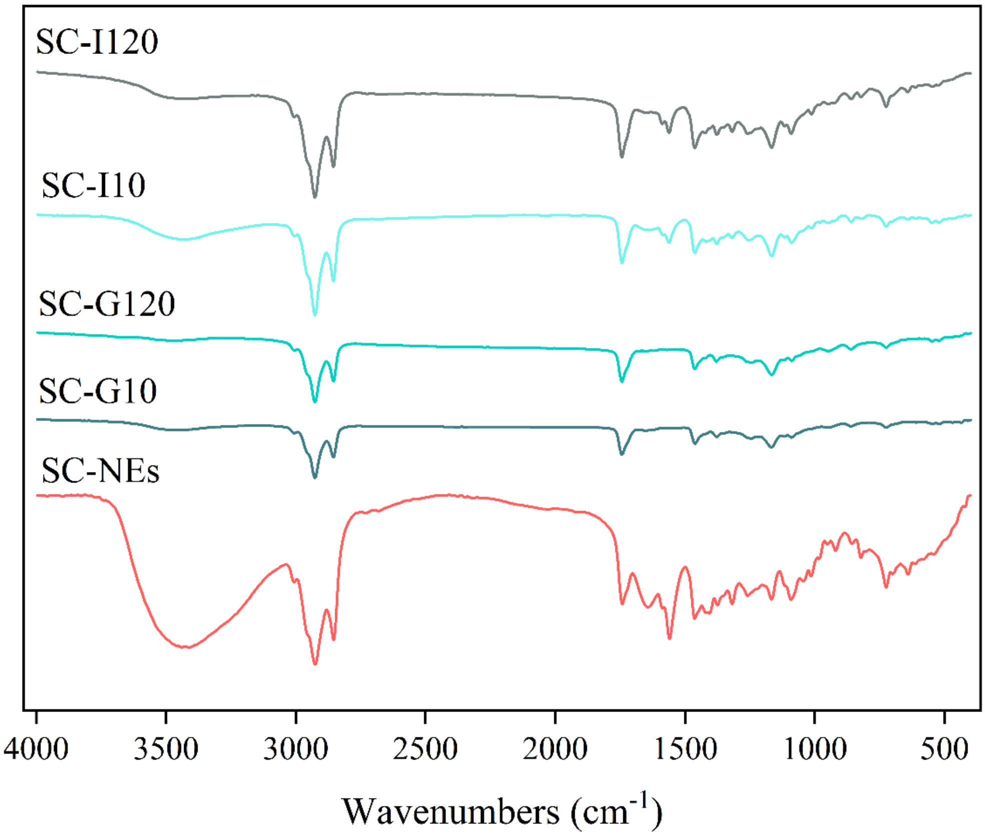
SC-I120
SC-I10
SC-G120
SC-G10
SC-NEs
4000
3500
3000
2500
2000
1500
1000
500
Wavenumbers (cm-1)

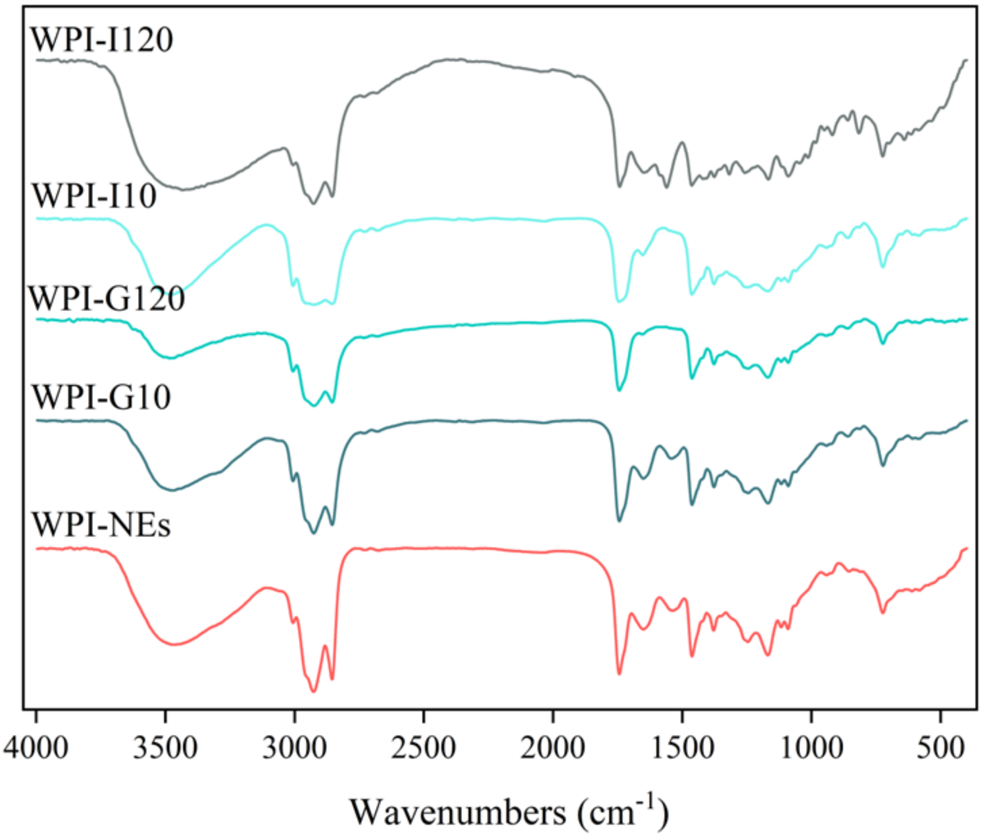
WPI-I120
WPI-I10
WPI-G120
WPI-G10
WPI-NEs
4000
3500
3000
2500
2000
1500
1000
500
Wavenumbers (cm-1)

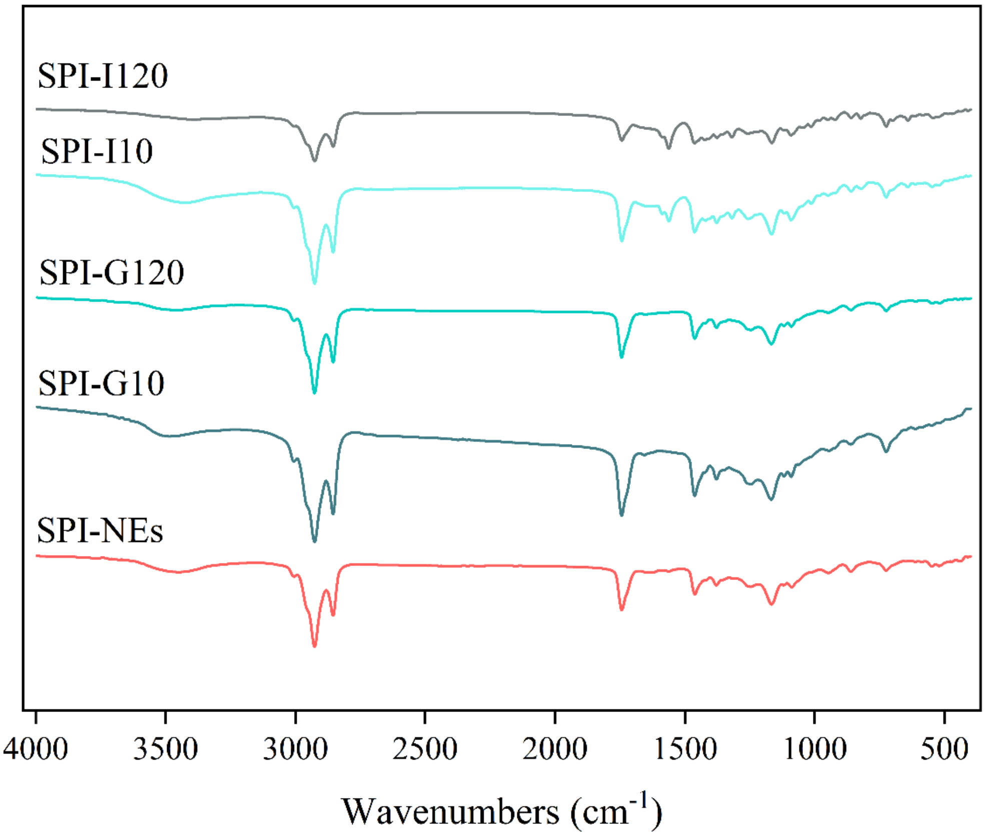

Figure.S2. FTIR spectra of WPI-, SC-, SPI -stabilized emulsions during in vitro digestion

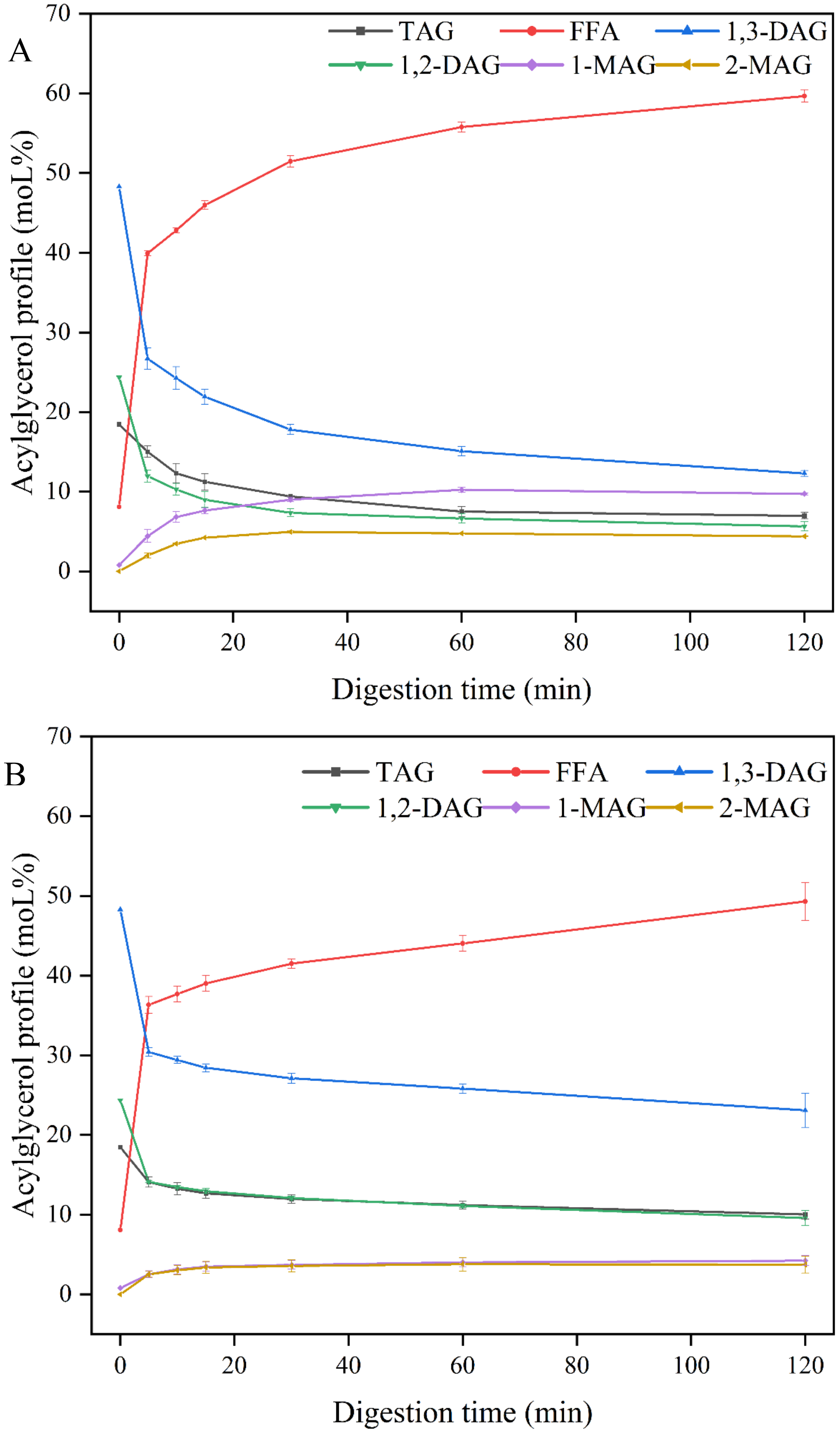
A
TAG
FFA
1,3-DAG
1,2-DAG
1-MAG
2-MAG
Acylglycerol profile (moL%)
Digestion time (min)
B
TAG
FFA
1,3-DAG
1,2-DAG
1-MAG
2-MAG
Acylglycerol profile (moL%)
Digestion time (min)

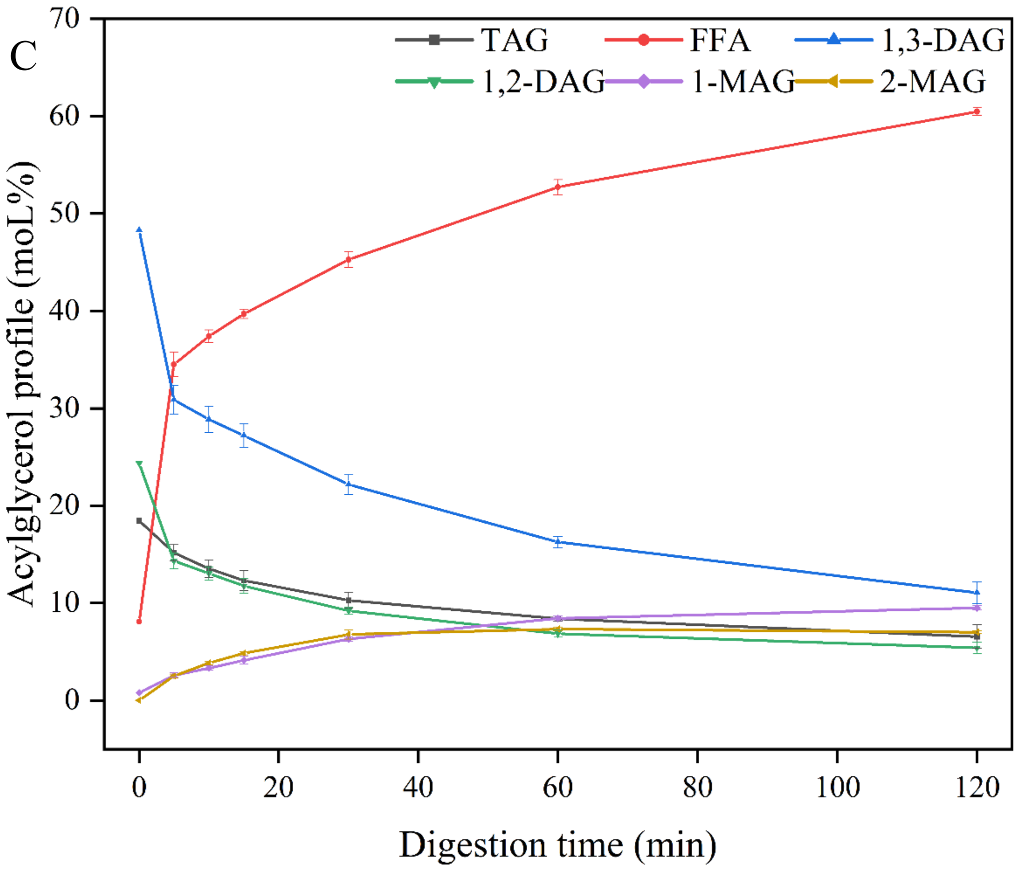

Figure.S3. Acylglycerol profiles of different protein-coated emulsifiers during intestinal digestion. The letters A - C represent the changes in acylglycerol profiles of WPI-, SC-, and SPI-coated DAG emulsions, respectively.
